# Hyperplasie congénitale des surrénales à révélation tardive: à propos d'un cas rare

**DOI:** 10.11604/pamj.2013.14.165.2186

**Published:** 2013-04-29

**Authors:** Sofia Jayi, Fatima Zahra Fdili, Hakima Bouguern, Hekmat Chaara, Abdelilah Melhouf

**Affiliations:** 1 Service de gynécologie-obstétrique 2, CHU HASSAN II de FES, Maroc; 2 Université sidi mohammed benabdellah, Maroc

**Keywords:** Hyperplasie congénitale des surrénales, déficit partiel en 21 hydroxylase, diagnostic, traitement, Congenital adrenal hyperplasia, 21 hydroxylase deficiency, diagnosis, treatment

## Abstract

L'hyperplasie congénitale des surrénales (HCS) par déficit en 21 hydroxylase à révélation tardive est une maladie à transmission autosomique récessive dont la présentation usuelle est une virilisation tardive para ou post pubertaire. Nous rapportons le cas d'une patiente âgée de 17 ans, ayant consulté pour aménorrhée primaire, chez qui l'examen clinique a trouvé un morphotype masculin, des signes de virilisation, des caractères sexuels secondaires présents avec une hypertrophie péniforme du clitoris. L’échographie pelvienne a confirmé la présence de l'utérus et des ovaires et a éliminé une cause tumorale ovarienne. Le dosage du sulfate de DHA (1145 ug/dl) et la 17 OH progestérone à 49,6 ng/ml étant élevés, ont orienté le diagnostic vers l'hyperplasie congénitale des surrénales à révélation tardive par déficit partiel en 21 hydroxylase. La TDM abdominale a trouvé une hypertrophie homogène bilatérale des surrénales. La patiente a été mise sous déxaméthasone 0,5 mg/ j avec déclenchement des cycles menstruels au bout de 4 mois de traitement. Une plastie clitoridienne ainsi qu'une étude moléculaire génétique étaient prévues mais la patiente était perdue de vue. A travers cette observation, et à la lumière d'une revue de la littérature, nous insistons sur les caractéristiques cliniques et para cliniques de cette entité, ainsi que l'intérêt du diagnostic précoce pour permettre une croissance normale, une puberté féminine et une fertilité satisfaisante.

## Introduction

Les hyperplasies congénitales des surrénales (HCS) sont des pathologies génétiques secondaires à un déficit de l'une des enzymes de la stéroidogénése. Nous rapportons un cas rare d'une hyperplasie congénitale des surrénales à révélation tardive par déficit partiel en 21 hydroxylase.

## Patient et observation

**Figure 1:**
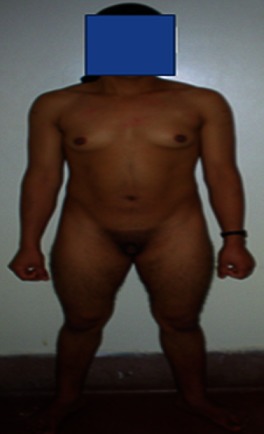
Morphotype masculin (hypertrophie musculaire, diamètre bi-acromial augmenté)

**Figure 2:**
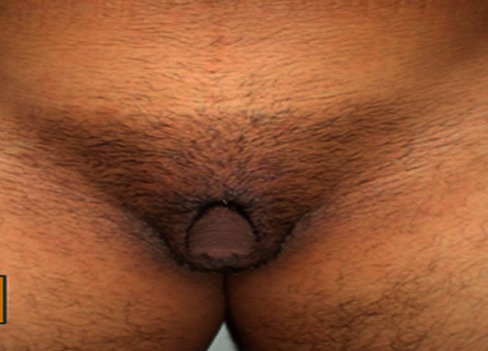
Pilosité de type masculine et hypertrophie clitoridienne péniforme

Mlle M.B âgée de 17 ans, a consulté pour aménorrhée primaire. L'examen clinique a trouvé un morphotype masculin (Figure 1) avec des signes de virilisation et des caractères sexuels secondaires présents: des seins développés mais avec une pilosité pubienne de type masculine et des organes génitaux externes ambigus: des grandes lèvres présentes avec une hypertrophie du clitoris péniforme (Figure 2). A noter que l'hymen et l'urètre étaient en place. L’échographie pelvienne a trouvé un utérus de petite taille environ 50 mm avec les 2 ovaires qui sont présents. Le dosage du sulfate de DHA (1145 ug/dl) et de la 17 OH progestérone (49,6 ng/ml) étant élevé a évoqué le diagnostic de l'hyperplasie congénitale des surrénales à révélation tardive. La TDM abdominale a trouvé une hypertrophie homogène bilatérale des surrénales. Ainsi la patiente a été mise sous déxaméthasone à raison de 0,5 mg/ j qui a permis le déclenchement des cycles menstruels 4 mois après le début du traitement. Une plastie clitoridienne ainsi qu'une étude moléculaire génétique étaient prévues mais la patiente était perdue de vue.

## Discussion

La fréquence de l'HCS est variable, elle touche 1 à 10% des femmes hyperandrogéniques en fonction de l'origine ethnique et géographique [1]. Elle est secondaire dans 90-95% des cas au déficit de la 21 hydroxylase en rapport avec des mutations du gène CYP21A2 [2] à transmission autosomique récessive [1].

**Figure 3:**
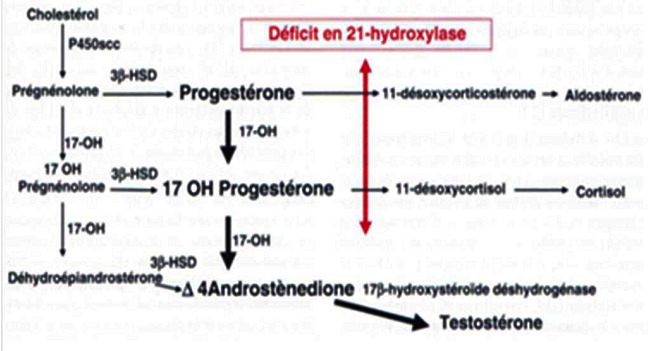
Biosynthèse des stéroïdes surrénaliens

La surrénale synthétise à partir du cholestérol et grâce à 5 enzymes, 3 hormones essentielles, cortisol, aldostérone et testostérone (Figure 3) [2]. L'absence de la 21 hydroxylase entraine l'augmentation de la sécrétion de la 17-OH progestérone et des androgènes surrénaliens [2].

Le déficit complet de cette enzyme est responsable de la forme dite complète de l'HCS (ambiguïté sexuelle à la naissance avec ou sans perte de sel selon le degré de déficit en aldostérone). Alors que le déficit partiel se traduit par un tableau clinique polymorphe [1, 3] survenant à l'enfance ou à l'adolescence, en rapport avec l'hyperandrogènie débutant en post-natal. Chez l'adolescente comme c'est le cas de notre patiente, les manifestations sont variables: l'hirsutisme qui apparait généralement en période péripubertaire est le signe le plus fréquent [2, 4], l'acné, l'alopécie, un morphotype androïde (hypertrophie musculaire chez une femme plutôt petite par soudure prématurée des cartilages de conjugaison, diamètre biacromial supérieur au diamètre bitrochantérien, une pilosité de type androïde). Une masculinisation des organes génitaux externes peut parfois se voir sous forme de clitoridomégali [1], Parfois péniforme comme le cas de notre patiente.

L'hyperandrogénie étant un facteur perturbateur de l'axe gonadotrope, sera à l'origine d'une dysovulation ou une anovulation se traduisant par des troubles du cycle, une aménorrhée ou encore une infertilité [5]. Cependant, il a été rapporté des cas asymptomatiques découverts à l'occasion d'enquête familiale [2, 6].

L’échographie pelvienne confirme la présence de l'appareil génital féminin et trouve fréquemment un aspect échographique d'ovaires micropolykystiques secondaire à l'hyperandrogènie qui ne devrait pas prêter à confusion avec le syndrome des ovaires micropolykystiques qui est un diagnostic d’élimination [7]. Le diagnostic biologique repose sur un dosage de base de la 17 OH progestérone supérieur à 2 ng/ml ou s'il est normal, une concentration > 10 ng /ml lors du test au synacthène [2, 8]. Les données concernant l'aspect des surrénales dans la forme tardive restent très ponctuelle elles sont en faveur d'une hyperplasie avec ou sans composante nodulaire [1]. Par ailleurs, certains diagnostiques différentiels doivent être bien connus de la part du gynécologue notamment les tumeurs virilisantes ovariennes ou surrénaliennes.

Le traitement dépend des symptômes, en cas d'hirsutisme une dose modérée d'hydrocortisone (30 mg par jour) ou de déxaméthasone (0,5 à 1 mg par jour) permet de freiner la sécrétion d'ACTH [1, 9]. L'acétate de ciprotérone peut aussi être utilisé en association avec l'ethinyl-aestradiol et semble être plus efficace en absence de contre-indication. Les troubles du cycle sont traités par hydrocortisone et ou progestatif. En cas d'infertilité les glucocorticoïdes améliorent voire même normalisent les cycles si non on passe au citrate de clomifène voir même l'assistance médicale à la procréation [2] Quant à l'anomalie des organes génitaux externes, la réduction de l'hypertrophie clitoridienne tout en préservant la sensibilité et les possibilités théoriques d’érection doit être précoce [9].

Le risque chez une femme ayant une mutation sévère sur l'un des 2 allèles d'avoir un enfant atteint d'une forme classique grave est de 0,4-2,5% selon les études et atteint 25% si le conjoint est lui aussi porteur d'une mutation sévère d'où l'intérêt de faire systématiquement une étude moléculaire du gène CYP21A2 chez la patiente et son conjoint et de la traiter par dexaméthasone à partir de la 8^ème^ SA si les mutations sévères sont présentent chez les 2 conjoints, afin de prévenir la virilisation d'un fœtus féminin [2]

## Conclusion

L'HCS à révélation tardive est l'un des diagnostic devant être systématiquement recherché par le gynécologue devant des patientes adolescentes voir même adulte présentant des signes d'hyperandrogènie avec virilisation associés ou non à des troubles du cycle ou à une infertilité. Le diagnostic doit être le plus précoce possible pour permettre une croissance normale, une puberté féminine et une fertilité satisfaisante. Pour ceci, l’étude moléculaire génétique systématique chez toute patiente ayant une forme tardive d'HCS et éventuellement de son conjoint ainsi que le diagnostic prénatal (notamment la biopsie trophoblastique) une fois la patiente est enceinte, sont d'un apport précieux [2].
